# Gingival Enlargement Associated with Orthodontics Appliance Increases Protein Carbonylation and Alters Phosphorylation of Salivary Proteome

**DOI:** 10.3390/dj12070208

**Published:** 2024-07-08

**Authors:** Zulieth Lopez Arrieta, Erika Rodríguez-Cavallo, Darío Méndez-Cuadro

**Affiliations:** 1 Faculty Health and Medical Science, University of Sinú, Cartagena 130014, Colombia; zuliethlopez@unisinu.edu.co; 2 Analytical Chemistry and Biomedicine Group, Exacts and Natural Sciences Faculty, University of Cartagena, Cartagena 130014, Colombia; erodriguezc1@unicartagena.edu.co

**Keywords:** gingival overgrowth, orthodontic appliance, protein carbonylation, phosphoproteins

## Abstract

Gingival enlargement is a common clinical sign in the gingival diseases associated with orthodontic treatment. Its biological mechanisms are not completely understood; nevertheless, the biochemical changes associated with these inflammatory and overgrowth processes could alter the post-translational protein modifications occurring in various locations within the mouth. Here, changes in the profiles of the carbonylated and phosphorylated proteins in saliva were examined in donors with gingival enlargement (seven men and seven women) and healthy donors (six men and eight women). The sociodemographic characteristics of both groups did not present significant differences. Carbonylation was measured by a quantitative immunoassay (Dot Blot), whereas the profiles of the phosphorylated proteins were visualized by SDS-PAGE with quercetin staining. Some phosphopeptides were also identified using a typical LC-MS-MS approach. Our results showed that gingival enlargement induced a significant increase in oxidative damage in salivary proteins. While a significant reduction in phosphorylation was observed at the stain level in SDS-PAGE, there was a slight increase in the number of phosphorylated proteins identified by MS in samples with gingival enlargement.

## 1. Introduction

Gingival enlargement (GE) is a common clinical sign of gingival pathologies and is a term used to describe the excessive growth of gingival tissue [1]. The global prevalence of GE is around 10%, and it is associated with systemic hormonal disorders and several blood dyscrasias, such as leukemia, thrombocytopenia, and thrombocytopathy, excluding the soft tissue lesions associated with fixed orthodontic treatment [2]. Depending on its extent and severity, GE may lead to functional disturbances such as altered phonetics, occlusion, and mastication, as well as aesthetic and psychological problems [3]. The biomolecular mechanisms of GE are not yet completely understood; however, it is usually considered to be the result of inflammation developed as a reaction to the accumulation of biofilm and possible overproduction of glycosaminoglycan-rich amorphous ground substances by fibroblasts [4]. In addition, recent studies have shown the association between gingival enlargement and the presence of matrix metalloproteins (MMP-8 and MMP-9) [5,6]

Some molecular events have been partially clarified in other clinical conditions characterized by inflammation, such as the association of the response and progression of the swelling of the affected tissue with the presence of post-translational modifications (PTMs) in its proteome [7]. PTMs are produced by the covalent attachment of specific chemical groups to the side chains of amino acids through enzyme-catalyzed or non-enzyme-catalyzed reactions, influencing both protein structure and cellular functions [8]. These changes also occur in normal physiological conditions and can be reversible, such as phosphorylation, or irreversible, such as carbonylation [9,10].

In general, PTMs are essential for regulating protein functions, and thus, the identification of modified proteins along with the metabolic networks they belong to is the first step in analyzing the dynamic changes arising from such networks at the cellular level. Although assessing the PTM patterns within protein molecules can substantially contribute to understanding the foundational mechanisms of a disease, their clinical implications still present significant challenges [11,12].

During oral inflammatory processes, an increase in protein carbonylation occurs because elevated protein carbonylation ensues due to the disparity between reactive oxygen species (ROS) production and the effectiveness of antioxidant defenses, known as oxidative stress. Likewise, alterations in the patterns of serine, threonine, and tyrosine phosphorylated residues are expected due to the signaling roles attributed to these PTMs [13,14]. 

Therefore, the inflammatory process in the oral cavity not only affects the normal salivary proteom, but it could provide an excellent sample to obtain new insights at the molecular level [15]. Furthermore, saliva is a preferred diagnostic body fluid because its collection is easy, non-invasive, and requires relatively simple instructions. For these reasons, the aim of this paper is to describe the post-translational changes, in terms of phosphorylation and carbonylation, that occur in the salivary proteome of patients with gingival enlargement associated with fixed orthodontic treatment to gain novel insights into the disease’s biological underpinnings.

## 2. Materials and Methods

This work was approved by the Ethics Committee No. 008 of 2020 at Universidad del Sinú. In accordance with the Declaration of Helsinki, each subject provided written consent.

### 2.1. Donors

**Figure 1:**
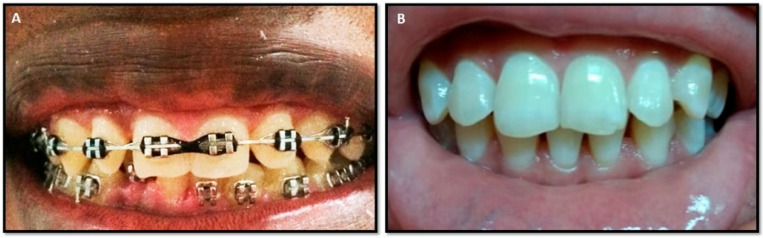
Clinical photography of study subjects. Panels showing the gingival state for two representative study subjects. (**A**) Study subject with gingival enlargement associated with orthodontic treatment. Note the overgrowth in lower anterior teeth (Grade II). (**B**) Healthy volunteer. Note the normal appearance of the gingiva (Grade 0).

The study involved 14 donors with gingival enlargement associated with orthodontic appliances (7 men and 7 women) and 14 healthy donors without orthodontic treatments and clinical evidence of inflammatory oral disease (6 men and 8 women). The classification of gingival enlargement was determined by the extent of overgrowth as follows: Grade 0 indicated no signs of gingival enlargement; Grade I denoted enlargement confined to the interdental papilla; Grade II involved enlargement of both the papilla and marginal gingiva; and Grade III indicated enlargement covering three-quarters or more of the tooth crown [16]. Fourteen subjects were diagnosed with Grade 0, eleven donors were diagnosed with GE Grade I, and three with Grade II (Figure 1). However, they were analyzed together as the group of donors with GE, with an average age of 19 ± 2.3. All donors received clinical treatment at the University of Cartagena. To strengthen the relationship between gingival enlargement and the presence of orthodontic appliances, the following inclusion and exclusion criteria were determined.

*Study Group:*

○ Young subjects between 17 and 22 years old.
○ Subjects with orthodontic appliances.
○ Subjects presenting some degree of gingival enlargement.

*Control Group:*

○ Young subjects between 17 and 25 years old.
○ Subjects without orthodontic appliances.
○ Subjects without gingival enlargement.

*Exclusion Criteria for Both Groups:*

○ Current or recent use of medications, such as anticonvulsants (e.g., phenytoin), immunosuppressants (e.g., cyclosporine), and calcium channel blockers.
○ Alcohol consumption.
○ Salivary dysfunctions.
○ O’Leary index greater than 20%.

### 2.2. Saliva Collection

The donors were asked not to eat, drink, or brush their teeth for two hours before the saliva collection. In addition, they were trained to collect their saliva from the floor of the mouth and then spit it into a sterile sampling bottle. This step was repeated until 5 mL of unstimulated saliva was collected from the recipient. The sample bottles were kept on ice throughout the collection process.

### 2.3. Extraction and Quantification of Salivary Proteins

The collected salivary samples were diluted with Milli-Q water at a 1:1 ratio and centrifuged at 3500 rpm, 4 °C, for 25 min to pellet any solid residue. The supernatant was obtained, mixed with 20% trichloroacetic acid (TCA) at a 1:1 ratio, and centrifuged at 5000 rpm for 30 min at 4 °C. The salivary protein pellet was purified using two rounds of 98% and 80% chilled acetone. The washed pellet was mixed with 10 μL of 2 M NaOH and suspended in lysis buffer (7 M urea, 2 M thiourea, 4% CHAPS). The protein concentration was determined using the Bradford method [17].

### 2.4. Determination of Phosphorylated Proteins’ Profiles

Phosphoproteins were visualized on 10% SDS-PAGE gel stained with quercetin, following the protocol described by Xi Wang in 2014 [18]. For this, 500 ng of salivary protein samples was loaded into each gel lane. Bovine serum albumin (BSA) and bovine milk caseins were included in the test as negative and positive controls of the phosphorylated proteins, respectively. The phosphoprotein bands were documented using excitation and emission wavelengths of 510 nm and 520 nm, respectively, in a ChemiDoc MP Imaging System^®^ (Bio-Rad, Hercules, CA, USA). Subsequently, the gels were washed with PBS (3 round for 5 min) and submitted to staining with Coomassie Brilliant Blue G-250 (Sigma-Aldrich^®^, St. Louis, MO, USA) to visualize the entire protein profile.

### 2.5. Quantitation of Carbonylated Proteins 

The dot blot immunoassay was used to quantify the oxidative damage [19] on the salivary proteins from donors with gingival enlargement associated with orthodontic appliances. For this reason, a calibration curve was built using BSA with different carbonyl index values, following a described protocol with slight differences. Briefly, the BSA solution (1 mg/mL) was divided into two equal parts. The first one was oxidized by treating with FeSO_4_ 10 mM at 37 °C for 2 h, and the second one was reduced with NaBH_4_, following standard protocols. The oxidized and reduced BSA solutions were dialyzed using a Dialysis Cassette, 7000 MWCO (Thermo Scientific^®^, Waltham, MA, USA), submerged in PBS 1 M [20].

Subsequently, the reduced and oxidized BSA solutions were incubated with mM DNPH in 0.5 M phosphoric acid at room temperature for 10 min. Finally, the DNPH-labeled BSA solutions were alkalized by strict incubation with NaOH 6 M for 10 min, and read at 450 nm [21]. The DNPH molar absorption coefficient (22,308 M^−1^ cm^−1^) was used to determine the nanomoles of carbonyl per milligram of protein; by stoichiometric mixing of the reduced and oxidized BSA solutions, the carbonyl index calibration curve was constructed.

Salivary proteins from the clinical samples and BSA working solutions were derivatized with DNPH following the protocol outlined by Levine et al. in 1990 for the dot blot assay [21]. Thus, 200 ng of each sample was manually spotted in triple doses onto PVDF membranes (Immun-Blot^®^ PVDF Membrane for Protein Blotting, Cat. #162-0177, Hercules, PA, USA). They were then incubated for 2 h at room temperature with 5% non-fat milk in PBS. For 2 h, the membranes were incubated with primary antibody Anti-DNP (Thermo Fisher Scientific, Mississagua, ON, Canada) at a dilution of 1:10,000 (Sigma).

Signals of the carbonylated proteins were developed using the HRP Chemiluminescent Substrate Reagent Kit (Novex^®^ ECL, PA, USA) for 2 min and captured in a ChemiDoc photodocumentation system (Bio-Rad). Image Lab software (Bio-Rad) was used to measure the optical density of each analyzed spot, maintaining a fixed circle area of 3.81 mm^2^. Intensity data were recorded in a matrix table in Microsoft Excel version 2016.

### 2.6. Identification of Phosphorylated Proteins by LC-MS

Imagines of the gel stained with quercetin and CBB brilliant were matched to select the phosphorylated protein bands. Thus, five bands were selected, excised from the gel, these five representative bands were numbered from 1 to 5 for the salivary proteins of subjects with gingival enlargement and from 6 to 10 for the salivary proteins of healthy subjects, and sent to the Proteomics Unit of Complutense University of Madrid to carry out gel trypsin digestion and an LC-MS/MS analysis, according their internal protocols. There, the protein bands were gel reduced with DTT, alkylated with iodoacetamide, and digested overnight at 37 °C with Trypsin sequencing grade at a 1/20 (*w*/*w*) ration [22]. Next, ZipTip C18 columns (Merck Millipore, Burlington, MA, USA) were used to desalt and concentrate the peptides, which were then eluted with 80% acetonitrile (MeCN)/0.1% trifluoroacetic acid, lyophilized in Speed-vac, resuspended in 2% MeCN and 0.1% formic acid, and stored at −20 °C until LC-MS/MS analysis.

The peptides were analyzed in an EASY-nLC 1000 System coupled to a Q-Exactive HF mass spectrometer with a Nano-Easy spray source (all from Thermo Scientific, Mississagua, ON, Canda). Using buffer A (mobile phase A: 2% acetonitrile, 0.1% formic acid), the peptides were trapped onto an Acclaim PepMap 100 Trapping column (Thermo Scientific, 20 mm × 75 m ID, 3 m C18 resin with 100 pore size). A 45 min gradient of 5% to 25% buffer B (100% acetonitrile, 0.1% formic acid) in buffer A and 10 min more of up to 35% buffer B at a constant flow rate of 250 nl/min was used.

For data acquisition, Q-Exactive HF was operated using an ion spray voltage of 1.8 kV and ion transfer temperature of 250 °C in a manner dependent on data acquisition (DDA) and in positive mode with Xcalibur 4.0 software. For the MS2 scan, the top 10 precursors with 2–6 charges were selected for energy dissociation (HCD) and fragmentation at 20 s intervals in the MS1 scan. The MS1 scan was acquired in the m/z range of 375–22,000 Da with 60,000 responses with a 3E6 gain control (AGC) target and a maximum ion time (ITmax) of 100 ms. The normal impact energy was 27%; resolved segments were evaluated across 30,000 channels with a target of 1E5 AGC at 50 ms ITmax.

For peptide identification, raw data were analyzed with the Mascot v. 2.6.1 search engine through the Protein Discoverer 2.2 Software (Thermo Scientific). SwissProt DB with taxonomic restriction (20,317 sequences, 21 February 2018) and a contaminant database with the sequences of the most common contaminants (247 sequences) were used to launch the search. The following parameters were chosen for the searches: tryptic cleavage after Arg and Lys, up to two missed cleavage sites allowed, and tolerances of 10 ppm for precursor ions and 0.1 Da for MS/MS fragment ions. Methionine oxidation and S, T, Y phosphorylation were set as variable modifications and cysteine carbamidomethylation was left fixed. A search against a decoy database (integrated decoy approach) was used to calculate the FDR. Mascot scores were adjusted with the filter algorithm. The acceptance criteria for protein identification were an FDR of <1% and the identification of at least one peptide with a high confidence (CI > 95%). The probability of the phospho site being in the peptide with this variant was estimated by the ptm-RS node, a tool in the Proteome Discovery 2.2 software, and the accepted probability was 75%.

### 2.7. Statistical Analysis

Sociodemographic data from the donors were analyzed using the measures of central tendency and dispersion. In this study, meticulous attention was devoted to validating the observations through the quantification of the salivary protein bands from the study participants (both healthy individuals and those with gingival enlargement) identified in SDS-PAGE, employing both quercetin and Coomassie staining techniques. To achieve this, five representative bands were meticulously selected and subjected to thorough analysis. Firstly, the bands were enumerated on the gels for each participant and categorized accordingly (bands 1 through 5). Subsequently, the relative frequencies for each band were computed utilizing Excel 2.0. Additionally, the band densities were determined by employing the ImageLab data analysis software package. Lastly, the intensity of the emitted fluorescence (density values) of each lane of the phosphorylation profile among the groups was compared and analyzed using the Wilcoxon test (*p* < 0.05). Meanwhile, the density values of the representative gel bands stained with Coomassie were analyzed using the *t*-test (*p* < 0.05). For the carbonylation results, data were only analyzed using the measures of central tendency and dispersion, since it was not possible to measure the identification signals for the proteins of the healthy volunteers under our conditions. The statistical analysis was performed using R statistical software. The mass spectrometry results were organized and detailed in a table.

## 3. Results

The salivary proteomes from the volunteer donors with GE due to orthodontic appliances and the healthy controls were obtained, quantified, separated by SDS-PAGE, and compared to evaluate the effects of GE on protein phosphorylation and carbonylation.

### 3.1. Determination of Phosphorylated Proteins’ Profiles

**Figure 2:**
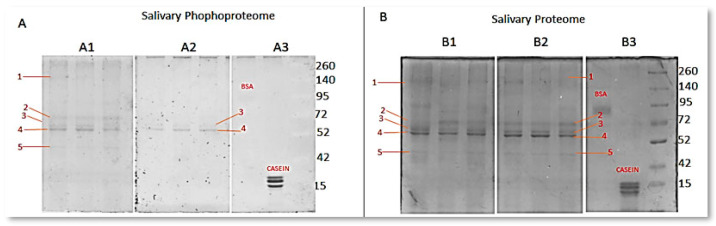
Profiles of phosphorylated and total salivary proteins in patients with gingival enlargement and healthy individuals by SDS-PAGE. Panel (**A**) shows the patterns of phosphorylated protein bands achieved with quercetin stain. Panel A1 shows the profile of phosphorylated salivary proteins in healthy subjects. Panel A2 shows the phosphorylated proteins found in saliva samples from subjects diagnosed with gingival enlargement. Panel A3 shows the controls for this experiment, with BSA as the negative control for phosphorylation and casein as the positive control for phosphorylation and molecular weight marker. In panel (**B**), the same gels depicted in panel (**A**) were subjected to Coomassie Blue Brilliant staining. This was conducted to obtain the profiles of total proteins and ensure uniform protein loading across all lanes. Panel B1 show the profile of salivary proteins observed in healthy volunteers. Panel B2 shows the profile of salivary proteins observed in patients with gingival enlargement. Panel B3 shows the phosphocontrol negative of BSA, phosphocontrol positive of Caseins, and molecular weight marker. Each lane contains 500 ng of protein from one individual. Note that five bands compatible with healthy volunteers and patients with gingival enlargement were identified.

**Figure 3:**
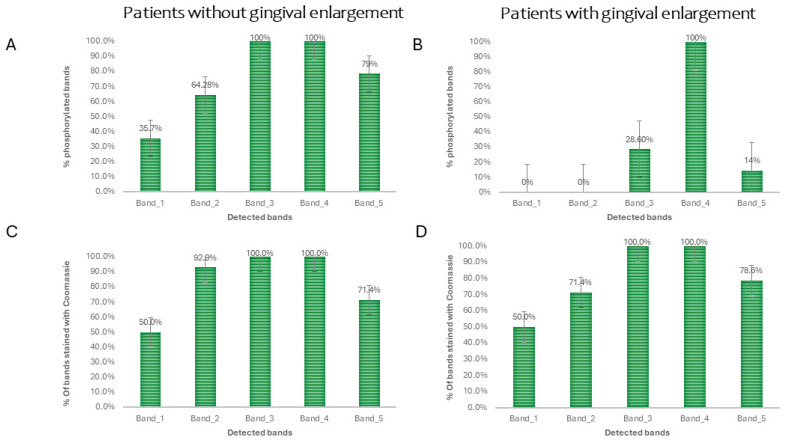
Frequency analysis of phospho-bands detected, and bands stained with Coomassie. Five protein bands were chosen from the profile obtained in Coomassie-stained SDS-PAGE. These bands were selected as references to determine which ones exhibited phosphorylation in the gel stained with quercetin. The number of bands present in each gel from each study subject was then counted and categorized, for both quercetin and Coomassie staining. Subsequently, the relative frequency for each of the 5 selected bands was determined. In panels (**A**,**C**), the relative frequency of phosphorylated protein bands achieved with quercetin and Coomassie stain, respectively, in samples from healthy volunteers is shown. The same order and description apply to panels (**B**,**D**) for patients with gingival enlargement. Note the significant changes in the relative frequency of bands detected in samples from patients with gingival enlargement.

A quantitative analysis of the phosphorylated protein bands in the control and case groups was performed from the electropherograms obtained. Qualitatively, it was found that the phosphorylated salivary protein profiles of the healthy controls were similar among them and exhibited five bands with different intensities (Figure 2). They showed two weak bands (bands 1 and 5) close to 140 kDa and 42 kDa, respectively, a moderate band at around 72 kDa (band 2), and two strong ones (bands 3 and 4) at 52 kDa. However, the salivary phosphoproteome obtained from the GE samples only showed two phosphorylated bands: a weak one (band 3) and the other moderate (band 4). This behavior was observed in all samples from the GE donors. The profiles were validated by the strong triple signal of phosphorylated caseins and the absence of signals for non-phosphorylated BSA. This description is detailed with the relative frequency in Figure 3.

**Figure 4:**
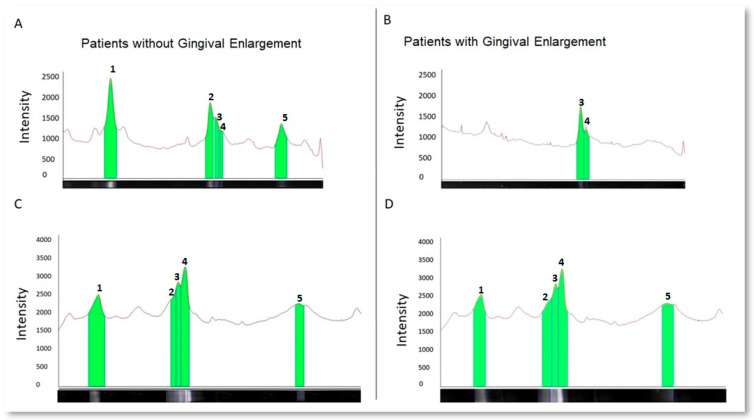
This figure was obtained by measuring the density of the 5 representative bands in the gels of salivary proteins from the study subjects with gingival enlargement and healthy individuals. Representative salivary protein densitograms. Curves in panels (**A**,**C**) show typical SDS-PAGE profiles of phosphorylated and total protein bands, respectively, from healthy donors. Peaks are numbered to indicate the intensity of the optical signal exhibited for each band. The same order and description are shown in panels (**B**,**D**) for samples obtained from patients with gingival enlargement.

From the densitograms obtained (Figure 4), a quantitative analysis was conducted to compare the levels of phosphorylation by evaluating each lane of the phosphorylation profile. The difference between groups suggested a greater number of phosphorylated proteins in patient samples without gingival enlargement.

**Figure 5:**
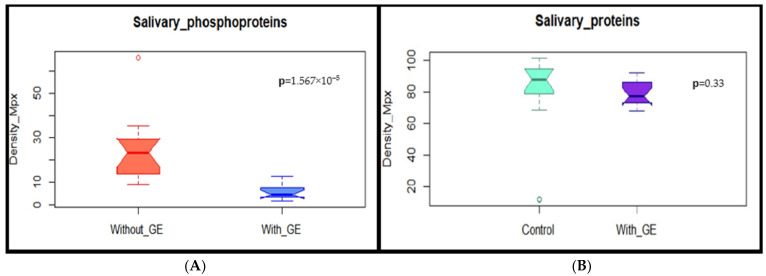
Box plots: (**A**) shows the density change in phosphorylation levels of salivary protein in patients with and without gingival enlargement, Wilcoxon test, *p*-value (1.567 × 10^−5^) (**B**) Shows the relative densities between the proteins of control volunteers and patients with gingival enlargement, obtained from gels stained with Coomassie, *t*-test, *p*-value (0.33).

When comparing the densitometry of the salivary proteins between the subjects with gingival enlargement and healthy subjects, results were obtained that confirm that the emissions recorded in the densitometry were not random, but rather the result of the selective labeling performed by quercetin on the phosphorylated proteins. The densities of the most representative bands (1, 2, 3, 4, and 5) stained with Coomassie were analyzed (*p* > 0.05). This showed that there was no significant difference in the loading of samples from the donors with and without gingival enlargement. The significant difference between the densitometries of gels stained only with quercetin (*p* < 0.05), suggests the presence of changes in the phosphorylation of salivary proteins between the healthy subjects and those with gingival enlargement. It was observed that phosphorylated bands were more frequent in saliva samples from the healthy subjects than in the patients with gingival enlargement (Figure 5).

### 3.2. Quantitation of Carbonyl Index by Dot Blot

**Figure 6:**
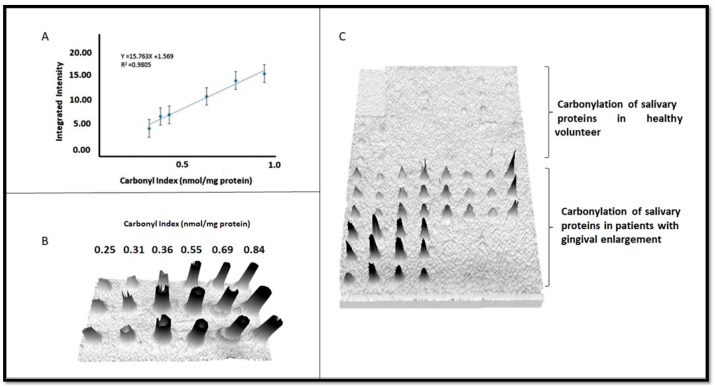
Dot blots to determine carbonyl indexes. (**A**) Panel A shows the linear regression curve obtained. (**B**) Panel B displays BSA spotted with carbonyl indexes in a range from 0.25 to 0.84 nmol of carbonyl/mg of protein. (**C**) An overview of the oxidation level for clinical samples spotted on PVDF membrane, showing differences between controls and GE patients.

The carbonyl index of the salivary proteins was quantified in samples from the donors with gingival enlargement. Moreover, identification signals for the proteins of the healthy donors were not possible (Figure 6). The measured values ranged from 0.38 to 0.69 nmol carbonyl/mg of protein. The results suggest that there was an increase in the carbonylation of salivary proteins in the donors with gingival enlargement associated with orthodontic treatment

### 3.3. Identification of Phosphorylated Proteins

To identify the phosphoproteins in saliva, the same five bands were excised from the CBB-stained gel from the donors both with and without gingival enlargement (Figure 3). Thus, a band between 250 and 140 kDa (band 1), three bands between 72 and 52 kDa (bands 2–4), and one band in the range of 52–42 kDa (band 5) were trypsin digested and analyzed by LC-MS-MS in a Q-Exactive HF mass spectrometer. In the results, 375 proteins were detected in the first band, 285 proteins in the second band, 265 proteins in the third band, and 249 and 372 proteins in the fourth and fifth bands, respectively. In Table 1, the phosphopeptides found in the protein bands obtained by SDS-PAGE are classified into three groups: phosphopeptides found in the saliva proteins from the healthy study subjects, phosphopeptides found in the saliva proteins from both groups of study subjects, and phosphopeptides found only in the study subjects with gingival enlargement.

## 4. Discussion

Post-translational modifications, such as phosphorylation and carbonylation, play important roles in protein function at the cellular level. In the case of protein phosphorylation, this is a crucial post-translational modification that impacts a wide range of cellular processes. The advent of recent biotechnologies has propelled the investigation of protein phosphorylation to become a major research focus. The study of the phosphoproteome in biological fluids presents an opportunity to uncover promising insights into novel systems related to protein function in health and disease [23]. Changes in the phosphoproteome have been associated with a wide variety of systemic diseases, including cancer, cardiovascular diseases, obesity and diabetes, and oral diseases such as dental caries [24,25,26,27,28]. The investigation of the phosphoproteins in saliva on a large scale marks the initial phase of a long-term endeavor aimed at identifying biomarkers for the diagnosis of both oral and systemic diseases [10]. Thus, the current study selected young subjects devoid of any preexisting systemic or oral conditions, aside from gingival enlargement, to mitigate potential confounding effects on protein outcomes and narrow down the relationship that may exist between gingival enlargement and the use of orthodontic appliances.

In this study, our objective was to explore the potential changes in the phosphoproteome induced by gingival enlargement associated with orthodontic appliances. We utilized SDS-PAGE in combination with quercetin staining to obtain a comprehensive profile of the salivary phosphoproteome. While SDS-PAGE coupled with Pro-Q Diamond is the most frequently utilized technique for detecting phosphorylated proteins, providing a simple and direct method that requires fewer complex processes, the quercetin method offers similar results at a lower cost [18]. In our study, we utilized gels configured with an excitation filter for Pro-Q Emerald 488 in a Chemidoc X System (excitation: 510 nm; emission: 520 nm long pass, BioRAd) for quercetin-stained gels. This differs from the method used by Xi Wang in 2014, who used a 498 nm long-pass emission filter along with a 365 nm laser excitation. We adjusted our results using measures obtained from negative and positive protein controls. Our findings suggested that gingival enlargement associated with orthodontic treatment induced an electrophorectic decrease in phosphorylation levels, potentially interfering with the proper functioning of proteins commonly phosphorylated [29].

While the presence of phosphorylated proteins does not directly indicate the health or disease state, their levels are influenced by factors such as age, genetics, overall health, and exposure to environmental changes. Despite this, phosphorylated salivary proteins are essential for maintaining oral health, as they act as inhibitors of bacterial growth, thereby preventing periodontal diseases and cavities. Proteins such as phosphorylated cystatin C or histatin have been associated with changes in oral health. Conversely, proteins like statherin are phosphorylated in response to inflammatory diseases of the oral cavity [30,31]. Table 1 elucidates the findings and phosphoproteome of healthy donors with gingival enlargement. Following analysis of the phosphoproteome, proteins exclusive to the saliva of patients with enlargement were identified. One such protein was the Beta-actin-like protein 2, a conserved protein linked to cellular motility. The phosphorylation of this protein may enhance cell mobility, reduce adhesion, and promote tissue growth processes. Notably, this protein has been suggested as a predictor for colorectal and ovarian cancer [32,33]. Another noteworthy protein is Lonf2, which is associated with the elimination of misfolded proteins and has been documented in the literature as a protein involved in quality control in senescence [34,35]. Its phosphorylation may suggest changes in its role in controlling the quality of structural proteins and its interaction with the proteasome in gingival tissue. On the other hand, oxidative stress is characterized by an imbalance between the production of free radicals and antioxidant levels, resulting in oxidative damage to molecules, including proteins. Markers of oxidative stress have been detected in saliva and have been associated with both systemic and local oral diseases, including inflammatory conditions such as gingivitis, periodontitis, and oral cancer, as well as Down’s syndrome and habits such as chewing khat or smoking cigarettes [36,37,38,39,40]. The presence of carbonylated proteins is commonly regarded as the most prominent marker of protein oxidation [41]. It is linked to clinical indicators of oral health. However, to the best of our knowledge, this study is the first to investigate carbonylated proteins in the saliva of donors with gingival enlargement associated with orthodontic appliances. In fact, our data reinforce previous works that have established a positive correlation among gingival enlargement caused by orthodontic appliances, the bioaccumulation of nickel, and increases in carbonylated proteins in gum samples [42].

The literature indicates that the level of salivary protein carbonyls is also influenced by both age and gender [43]. Based on the aforementioned observation, we opted to select two study groups with comparable distributions of socio-demographic characteristics. Notably, in this study, no significant differences were observed in the quantified carbonylation of salivary proteins between men and women (*p* > 0.05). Additionally, we deliberately chose donors without other pathologies to mitigate potential false positives, as oral or systemic diseases have been known to elevate the levels of carbonyls in salivary proteins [44,45]. The circadian cycle not only influences the profile of salivary proteins, but also affects various physiological processes within the body. Moreover, it was demonstrated by Haixiang Su et al. (2007) that the circadian cycle induces significant changes in the carbonyl quantification in saliva [46]. This finding influenced our decision to collect samples between nine and eleven in the morning for all donors in the study, ensuring that salivary protein oxidation was independent of factors that could potentially bias the data. These results underscore the importance of further investigating the molecular changes associated with gingival enlargement to gain a deeper understanding of the underlying processes and develop improved methods for diagnosis and treatment.

Our research indicates that gingival enlargement impacts the levels of carbonyls and phosphate groups in saliva proteins. Carbonylated proteins and phosphoproteins in saliva appear to serve as alternative biomarkers of gingival enlargement. However, the mechanisms underlying protein carbonylation and phosphorylation in relation to gingival diseases remain unclear, and further investigation is needed to elucidate the relationship between post-translational modifications in saliva proteins and oral pathologies.

## Figures and Tables

**Table 1:** Phosphorylated peptides and proteins identified.

N°	Master Protein Accessions	Master Protein Descriptions	Gel Band	Start–End	Phospho-Residue	Other Modification	Function *
A. Phosphoptides from healthy subjects							
1	P04745; P19961	Alpha-amylase 1/Alpha-amylase 2B	9	319–352	GHGAGGASILTFWDARL**Y(351)**KMAVGFMLAHPYGFTR	1xOxidation [M25]	Hydrolysis of (1→4)-alpha-D-glucosidic bonds in polysaccharides comprising three or more (1→4)-alpha-linked D-glucose units
2	Q1L5Z9	LON peptidase N-terminal domain and RING finger protein 2	6	490–497	L**S(491)**ELLASR	1xPhospho [S2(100)]	It is believed to activate protein ligase activity. It is estimated to be located in the cytoplasm
3	Q8NHR7	Telomere repeats-binding bouquet formation protein 2	6	184–214	KFLGELHDFIPGTSGYLAYHVQNEINMSAIK	2xPhospho [T/S/Y]	Meiosis-specific telomeres are associated with proteins located in meiotic telomeres and the inner nucleus; this is an important step in homologous synthesis and synapsis
4	Q9Y694	Solute carrier family 22 member 7	6	49–71	CALPGAPANF**S(59)**HQDVWLEAHLPR	1xCarbamidomethyl [C1]; 1xPhospho [S11(100)]	Isoform 2 acts as a bidirectional multispecific transporter independent of Na+
5	Q01518	Adenylyl cyclase-associated protein 1	9	295–312	SGPKPFSAPKPQTSPSPK	1xPhospho [S/T]	It directly controls filament strength and plays a role in many developmental processes and morphologies, including mRNA localization and establishment of cell polarity
B. Phosphoptides from subject with GE							
6	P04745; P19961	Alpha-amylase 1/Alpha-amylase 2B	4	1–35	MKFFLLLFTIGFCWAQYSPNTQQGRTSIVHLFEWR	1xAcetyl [N-Term]; 1xCarbamidomethyl [C13]; 1xPhospho [T/S/Y]; 1xOxidation [M1]	Hydrolysis of (1→4)-alpha-D-glucosidic bonds in polysaccharides with three or more (1→4)-linked D-glucose units
7	P60712; P68032	Actin, cytoplasmic 1/Actin, alpha cardiac muscle 1	5	51–61	DS**Y(54)**VGDEAQSK		Actin is a protein that is highly conserved and polymerizes to form filamentous networks within the cytoplasm of cells
8	Q562R1	Beta-actin-like protein 2	6, 10	317–327	EII**T(320)**LAPSTMK	1xPhospho [T4]; 1xOxidation [M10]	Actin is a versatile protein that plays an important role in many cellular processes and is found in all eukaryotic cells
9	Q07973	1,25-dihydroxyvitamin D(3) 24-hydroxylase, mitochondrial	1	252–280	MMV**T(255)**PVELHK**S**L(262)N**T(264)**KVWQDH**T(271)**LAWDTIFK	4xPhospho [T4; S11; T14; T21]; 2xOxidation [M1; M2]	Cytochrome P450 monooxygenase, vital for catabolism and vitamin D without calcium homeostasis
10	Q7RTU9	Stereocilin OS=Homo sapiens	1	913–927	SLVNQSVQDGEEQVR	1xPhospho [S]	It is necessary for the development of bilateral connections between the stereocilia of outer hair follicles that are involved in cell function, biogenesis, and response to stimuli
11	Q8IZL9	Cyclin-dependent kinase 20	1	94–113	HAQRPLAQAQVK**S(106)Y(107)**LQMLLK		Together with TBC1D32, it regulates primary ciliary formation by connecting the ciliary membrane assembly to the axoneme and makes GLI2 productive in response to SHH signaling. It also plays a role in cell growth and activates CDK2, a key kinase in cell regulation, through phosphorylation of the threonine residue ‘Thr-160′
12	Q12906	Interleukin enhancer-binding factor 3	4	128–143	VADNLAIQLAAV**T(140)**EDK		RNA-binding protein that plays an essential role in the biogenesis of circular RNAs (circRNAs). Participates in a wide range of transcriptional and post-transcriptional processes. Binds to poly-U elements and AU-rich elements (AREs) in the 3′-UTR of target mRNAs
13	Q9NTX9	Protein FAM217B OS=Homo sapiens	4	300–316	S**T(301)**KLQRWDL**S(309)**GSGSSSK		N/A
C. Phosphoptides from both subject groups							
14	P04745; P19961	Alpha-amylase 1/Alpha-amylase 2B	1, 3, 4, 8, 9	158–173	TGSGDIEN**Y(166)**NDATQVR		RNA binding proteins are crucial for RNA biogenesis (circRNA) and participate in various transcriptional and post-transcriptional processes. It binds to poly-U elements and AU-rich elements (AREs) found in the 3′ UTR of mRNAs
15			2, 3, 4, 8, 9	108–139	IYVDAVINHMSGNAVSAGTSSTCGSYFNPGSR	1xCarbamidomethyl [C23]; 1xPhospho [T/S]; 1xOxidation [M10]	
16	P13796	Plastin-2	1, 2, 6, 7	4–15	G**S(5)**VSDEEMoMoELR	2xOxidation [M8; M9]	An actin-binding protein involved in activating T-cells in response to co-stimulation via TCR/CD3, CD2, or CD28. It regulates the cell surface expression of IL2RA/CD25 and CD69
17	P42680	Tyrosine-protein kinase Tec	3, 4, 8, 9	569–580	YTNYEVVTMVTR	1xPhospho [Y/T]; 1xOxidation [M9]	A non-receptor tyrosine kinase involved in signal transduction from various receptors, contributing to multiple downstream pathways, including the regulation of the actin cytoskeleton
18	P60712; P68032	Adenylyl cyclase-associated protein 1	5, 10	360–372	QEYDESGPSIVHR	1xPhospho [Y/S]	Directly influences filament dynamics and is involved in numerous intricate developmental and morphological processes, such as mRNA localization and the establishment of cell polarity

* It describes the phosphorylated proteins in healthy study subjects and those with gingival enlargement, indicating the phosphorylated residues and their biological functions. Functions annotated were taken from the uniprot data base) Highlighted in bold appears the phosphorylated residues in the peptide.

## Data Availability

The data presented in this study are available on request from the corresponding author.
